# Microalgal Carotenoids: Therapeutic Application and Latest Approaches to Enhance the Production

**DOI:** 10.3390/cimb44120427

**Published:** 2022-12-09

**Authors:** Priyanka Sirohi, Hariom Verma, Sandeep Kumar Singh, Vipin Kumar Singh, Jyoti Pandey, Saksham Khusharia, Dharmendra Kumar, Kaushalendra, Pratibha Teotia, Ajay Kumar

**Affiliations:** 1 Department of Biotechnology, Noida International University, Greater Noida 203201, India; 2 Department of Botany, B.R.D. Government Degree College Duddhi, Sonbhadra 231216, India; 3 Division of Microbiology, Indian Agricultural Research Institute, Pusa, New Delhi 110012, India; 4 Department of Botany, K.S.Saket P.G.College, Ayodhya 224134, India; 5 Department of Biochemistry, Singhania University, Pacheri Barı, Jhunjhunu 333515, India; 6 Kuwar SatyaVira College of Engineering and Management, Bijnor 246701, India; 7 Department of Zoology, C.M.B. College, Deorh, Ghoghardiha 847402, India; 8 Department of Zoology, Mizoram University (A Central University), Pachhunga University College Campus, Aizawl 796001, India; 9 Department of Postharvest Science, Agricultural Research Organization (ARO)—Volcani Center, Rishon Lezion 7505101, Israel

**Keywords:** microalgae, carotenoids, antioxidant properties, optimization of cultures, synthetic biology, metabolic engineering

## Abstract

Microalgae are microscopic photosynthetic organisms frequently found in fresh and marine water ecosystems. Various microalgal species have been considered a reservoir of diverse health-value products, including vitamins, proteins, lipids, and polysaccharides, and are broadly utilized as food and for the treatment of human ailments such as cancer, cardiovascular diseases, allergies, and immunodeficiency. Microalgae-derived carotenoids are the type of accessory pigment that possess light-absorbing potential and play a significant role in metabolic functions. To date, nearly a thousand carotenoids have been reported, but a very less number of microalgae have been used for the commercial production of carotenoids. This review article briefly discussed the carotenoids of microalgal origin and their therapeutic application. In addition, we have briefly compiled the optimization of culture parameters used to enhance microalgal carotenoid production. In addition, the latest biotechnological approaches used to improve the yields of carotenoid has also been discussed.

## 1. Introduction

Due to depleting natural resources and an increasing global population, there has been significant growth in the quest for bioactive substances and alternative food sources in recent decades. However, due to the rise of new diseases and the negative side effects of using allopathic medicine to treat a variety of human ailments, it was urgent to look for an affordable, all-natural replacement [1,2,3]. Microalgae are chosen in this regard as a source of alternative foods or pharmaceutically necessary substances, and they are receiving a lot of attention. Therefore, the scientific community is continuously exploring the hidden potential of microalgal products as an alternative to pharmaceuticals and food products [2]. These microalgal groupings are thought to have existed in the aquatic ecosystem for more than 3.5 billion years [4]. Nearly 8000 species of diverse microalgae have been recorded from a variety of natural habitats, including hostile ones, and new species are still being discovered and classified [5]. Microalgae, the small photosynthetic organisms of both prokaryotic and eukaryotic nature, found in terrestrial and aquatic environments, simply require water, carbon dioxide, and sunlight for growth [6]. The characteristic plasticity in the growth of microalgae under autotrophic, heterotrophic, and mixotrophic conditions has offered multiple opportunities for human welfare [7]. Diverse microalgal groups with considerable economic importance are reported largely from classes including chlorophyceae, cyanophyceae, dinophyceae, rhodophyceae, and bacillariophyceae [6]. All the known classes of microalgae differ with respect to the nature of reserved food material, pigmentation, cell wall composition, flagellation, and mode of reproduction [8]. Moreover, the characteristics composition of microalgal genera within classes varies with respect to total protein, carbohydrate, and lipid content [9].

Microalgae are recognized as a superfood for people since they are an excellent source of nutraceuticals and necessary medicinal chemicals [2]. Foods with a high concentration of antioxidants, fiber, minerals, vitamins, and a distinctive nutritional profile are referred to as “superfoods” [10]. Currently, a wide range of microalgal products such as pigments, proteins, vitamins, polysaccharides, and lipids are available in the global market as high-value health products. More than 19,000 t of microalgal biomass generating about USD 5.7 billion yearly in leading countries such as Taiwan, the United States, Japan, etc., are reported [1,11,12]. Microalgae possess several advantages over higher plants and organisms as they can be grown on non-arable land and have a higher survival rate under harsh environmental conditions. In addition, they have the potential to acclimatize rapidly under given environmental conditions [13,14]. Different microalgal species, including *Chlorella vulgaris*, *Spirulina platensis*, *Haematococcus pluvialis*, and *Dunaliella salina*, have been described by a number of authors as being an important source of pigments, including carotenoids as well as vitamins, antioxidants, and anti-cancerous compounds [15,16,17]. Currently, pharmaceutics, colors, meals, and cosmetics are some of the industries using these microalgal products as a resource material [18].

Therefore, this review article has been compiled to briefly discuss the carotenoids of microalgal origin and their therapeutic application. In addition, we have briefly compiled the optimization of different culture parameters helpful in enhancing microalgal carotenoid production. Finally, the review has also discussed the latest biotechnological approaches to improve the yields of desired carotenoids.

## 2. An Overview of Microalgal Carotenoids

In microalgae, various pigments such as carotenoids, chlorophyll, and phycobilins, xanthophylls are present, which play a crucial role in normal physiology such as photosynthesis as light harvesting and energy transfer molecules. Microalgal pigments possessing complex molecular structures are reported to absorb different wavelengths of light [19,20,21]. Usually, it has been considered that the content of carotenoids and chlorophyll in the microalgae is greater than in the higher plants [19].

Carotenoids are tetraterpene derivatives, fat-soluble pigment molecules having a 40-carbon polyene structure and the ability to absorb light with a wavelength of 400–550 nm that is beneficial for photosynthesis. Even though carotenoids have a linear chain structure, they also include conjugated double bonds. However, photosynthetic organisms such as microalgae have cyclic ring-like structures at the terminal position. Oxygen atoms are also present in the ring structure as hydroxyl or epoxide groups. Carotenoids typically have well-defined three-peaked absorption spectra, although epoxidation can change the absorption maxima depending on where double bonds are located in the ring [22,23,24].

According to estimates from various archaea, algae, plants, and bacteria, approx. 1100 distinct natural carotenoids have been identified [25,26,27]. Numerous carotenoids, including β-carotene, lutein, zeaxanthin, astaxanthin, canthaxanthin, and fucoxanthin have been reported in different microalgae [28]. The details chemical structures of carotenoids have been presented in Table 1.

These carotenoids are classified into oxygen-containing xanthophylls such as astaxanthin and zeaxanthin and without oxygen-containing, such as β-carotene and lycopene. Carotenoids are divided into two classes based on how they act in photosynthesis, though. The principal carotenoids, β-carotene, and lutein are crucial for the energy transfer to chlorophyll, which aids in photosynthesis and preserves cellular viability. Astaxanthin and canthaxanthin, two secondary carotenoids, on the other hand, operate as protective components in stressful situations by building protective layers and shielding cells from oxidative stress [29]. 

Carotenoids function as quenchers of reactive oxygen species (ROS), serving as antioxidant molecules and shielding the cells and tissues from oxidative damage. They are also engaged in the oxygen and thermal dissipation of excess energy in photosynthetic machinery [30,31,32]. Although the concentration of carotenoids in the microalgal groups is normally low (0.5% g/dry weight), it has been shown that some species, such as *Dunaliella salina*, can accumulate up to 10% g/dry weight under unfavorable conditions [33]. Details of different microalgal species producing carotenoids, geographical locations, yield, and important properties are listed in Table 2

### Biosynthesis of Carotenoids

**Figure 1:**
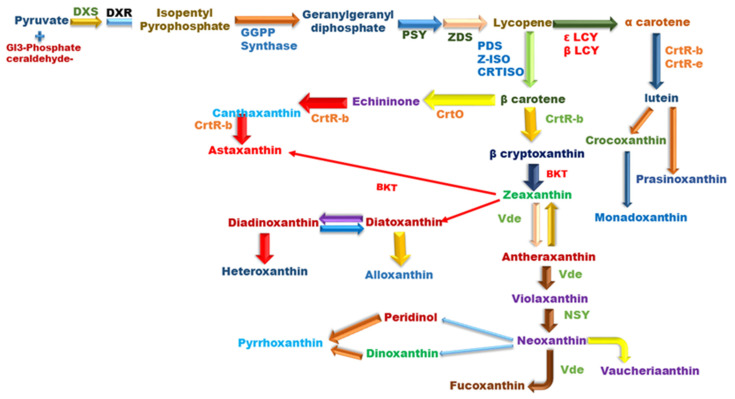
The overview and schematic representation of carotenoid biosynthesis, Figure, is adapted and modified from Gong and Bassi [34]. (Major enzymes involved during microalgae carotenogenesis are: ζ-carotene desaturase (ZDS), ζ-carotene isomerase (Z-ISO), Deoxy-D-Xylulose-5-phosphate Reductoisomerase (DXR), Carotenoid Isomerase (CRTISO), violaxanthin de-epoxidase (VDE), ε-cyclase (ε-LCY), β-carotene ketolase (BKT), DXP synthase (DXS), β-cyclase (β-LCY), GGPP synthase (GGPS), phytoene desaturase (PDS), phytoene synthase (PSY), β-carotene hydroxylase (CrtR-b), ε-carotene hydroxylase (CrtR-e), β-hydroxylase (CHYβ), neoxanthin synthase (NSY), carotene β-ketolase (CrtO/CrtW), carotene β-hydroxylase (CrtR-β)).

Carotenoids belong to the isoprenoid subfamily, and their biosynthetic pathway varies in different organisms. However, it utilizes a comparable metabolic route, where dimethylallyl diphosphate or isopentenyl diphosphate serves as the precursor and is produced either from acetyl Co-A to dimethylallyl diphosphate or from glyceraldehyde-3-phosphate and pyruvate. Nevertheless, microalgae generally follow the methylerythritol-4-phosphate pathway during synthesis [46]. Deoxyxylulose 5-phosphate synthase performs enzymatic catalysis to make deoxyxylulose 5-phosphate, which is then further reduced to methylerythritol-4-phosphate by deoxyxylulose 5-phosphate reductoisomerase during the biosynthetic route [47]. The schematic representation of carotenoid synthesis is presented in Figure 1.

After synthesis, isopentenyl diphosphate isomerizes to dimethylallyl diphosphate, which further condenses to polyprenyl pyrophosphate followed by head-to-tail condensation through prenyltransferases. Condensation of C-5 units leads to the formation of geranylgeranyl pyrophosphate, which further condenses to yield “phytoene” by enzyme phytoene synthase and acts as a rate-determining step during carotenoid production. In the presence of phytoene desaturase, phytoene is converted to ζ-carotene to produce pro-lycopene, which is further isomerized to lycopene using carotenoid isomerase. However, after lycopene synthesis, it is bifurcated firstly to β-carotene through cyclization by the action of lycopene β-cyclase and undergoes hydroxylation reaction through carotene β-hydroxylase to yield zeaxanthin, later transformed to violaxanthin by the help of epoxidation that depends on the oxygen and various co-factors such as ferredoxin, NADH, and FAD [48]. Under high light intensity, violaxanthin is converted back to zeaxanthin through de-epoxidation by the action of violaxanthin de-epoxidase. However, in another bifurcated branch, β-cyclase and ε-cyclases both act in a coordinated fashion to yield α-carotene which is further hydroxylated to lutein by the action of carotene β-hydroxylase.

## 3. Therapeutic Application of Carotenoids

Scientists have recently looked into how carotenoids may be used to treat a variety of medical conditions. As a result, many synthetic carotenoids produced through processes such as condensation, isomerization, and dehydration of carbonyl compounds are now available on the market. Typically, two phosphonium salts and one dialdehyde molecule are combined to create chemical carotenoids (Witting processes), which are then isomerized to create carotenoids such as astaxanthin, β-carotene, and lycopene [49,50]. In earlier research, many authors noted the positive effects of carotenoids generated from microalgae on health.

### 3.1. Anticancerous Activity

The anti-cancer properties of carotenoids have been proven in several in vitro and in vivo investigations. These research findings suggest that carotenoids might protect against a variety of human malignancies, including breast, intestinal, leukemic, lung, oral, and prostate cancer [51,52]. However, carotenoids induce limited cell proliferation as part of their anti-cancer action, among other ways. In previously published studies, various authors have reported the anti-cancerous properties of β-carotene, fucoxanthin, astaxanthin, lutein, and zeaxanthin [51]. During the study, authors have reported most common mechanisms followed by carotenoids are cell cycle arrest, apoptosis, and metastasis [52]. Their research revealed the role of these carotenoids in reducing the size and frequency of liver neoplasias. A study by Kumar et al. [52] reported the anti-cancerous properties of fucoxanthin. According to Jyonouchi et al. [53], astaxanthin can help shrink tumors and lighten their burden.

### 3.2. Anti-Diabetes Activity

Recent research on carotenoids strongly implies their potential in treating and managing diabetes. Carotenoids have been shown to lower the incidence of type 2 *diabetes mellitus* development in human beings. The intake of carotenoids has also been found to be negatively correlated with glucose levels [54,55]. In previous studies, the antagonistic relationship between β-carotene and fasting blood sugar levels has been observed [5]. Eating foods rich in carotenoids may lower the chance of developing type 2 diabetes [55]. Zeaxanthin, lycopene, and lutein are said to help prevent diabetic retinopathy, according to Brazionis [56]. Astaxanthin’s use in treating diabetes was described by Yeh et al. in their study [57]

### 3.3. Anti-Inflammatory

The immune system’s reaction to acute inflammation and infection involves the activation of defensive mechanisms against foreign substances and organisms. In order to treat the injury and inflammation, proinflammatory mediators should be suppressed, and antiinflammatory mediators should be activated [58]. However, the anti-inflammatory actions of carotenoids have also been reported by various authors [58,59]. Utilizing carotenoids helps to regulate chronic inflammatory diseases by considerably inhibiting the synthesis of pro-inflammatory cytokines, nitric oxide (NO), and prostaglandins [59]. Bennedsen et al. [60] reported the role of astaxanthin in reducing gastric inflammation. Similarly, Macedo et al. [61] reported that the treatment with 5 mM astaxanthin significantly improved the phagocytic activity of neutrophils. Carotenoids such as β-carotene possess antioxidant properties which hamper the activity of free radicals in the digestive tract by many folds [62,63]. In addition, it also enhances metabolic activity and regulates the synthesis of rhodopsin [64]. Therefore, also referred to as a provitamin and taken as a supplement [65]. Other carotenoids such as zeaxanthin and lutein have found their application in nutraceuticals and play a crucial role in maintaining the eye’s health as an essential pigment for the macula [66]. Other carotenoids such as fucoxanthin have also shown anti-cancerous and antitumor activity [67].

### 3.4. Antioxidant Activity of Carotenoids

The normal metabolic process in the living organism leads to the generation of free radicals such as H_2_O_2_^•−^, OH^•−^, and O_2_^•−^; these free radicals are also referred to as reactive oxygen species (ROS). However, these free radicals operate as a signaling molecule at lower concentrations and are crucial for maintaining cellular homeostasis and function [68,69]. The antioxidative enzymes typically neutralize these free radicals. However, due to the high reactivity of these free radicals, an excess of them can lead to oxidative stressors, which can induce the denaturation of enzymes, lipid peroxidation of cell membranes, and denaturation of nucleic acids. In addition to cancer, it also results in metabolic and physiological issues [70,71,72].

Carotenoids are very diverse and one of the largest groups of pigments possessing potent antioxidant activity and acting as quenchers of free radicals. In addition, it prevents free radical formation by ceasing oxidation reactions [73,74,75]. As microalgal groups grow under strong sunlight and have high oxygen concentrations, which favor the formation of free radicals, the characteristics of antioxidants, in fact, depending on the connected functional groups and conjugated double bonds [76]. The microalgae’s outstanding antioxidant capability was demonstrated by their average growth and lack of oxidative damage [77].

In the microalgae, astaxanthin, fucoxanthin, β-carotene, zeaxanthin, and violaxanthin are the most common carotenoids and possess antioxidant properties. In the last few years, various microalgal strains have been reported that contain a significant amount of carotenoids. For instance, *Dunaliella salina* has been reported as an excellent source of β-carotenes [78,79,80]. Nobre et al. [81] and Ceron et al. [82] reported that strain *Haematococcus pluvialis* might be a potential source of β-carotene, lutein, and canthaxanthin. Macías-Sánchez et al. [83] reported *Scenedesmus almeriensis* as a source of lutein and β-carotene. Similarly, Ahmad et al. [84] reported *Chlorella vulgaris* as a potent source of canthaxanthin and astaxanthin.

The antioxidant potential of microalgal carotenoids and their methods of action have been documented by a number of authors [85,86]. For instance, astaxanthin reduces the generation of intracellular ROS by altering the enzymes that respond to oxidative stress and by suppressing the Sp1/NR1 signaling pathway, which is an indication of oxidative stress [87,88]. According to Xue et al. [89], astaxanthin promoted the production of Nrf2 and Nrf2-targeted proteins and increased the number of antioxidant enzymes such as superoxide dismutase, catalase, and glutathione peroxidase. Similar to other carotenoids, fucoxanthin has recently been noted by a number of scientists to have the strong antioxidant potential [40]. The antioxidant capacity of *Porphyridium cruentum*, *Phaeodactylum tricornutum*, and *Chlorella vulgaris* ethanolic extracts by the β-carotene-linoleate model was reported by Rodriguez-Garcia et al. in a study [90]. All of these strains had antioxidant activity, although the strain *C. vulgaris* displayed a higher concentration of BHA and BHT (butylated hydroxytoluene). Similar to this, Murthy et al. [91] showed that -carotene isolated from *Dunaliella salina* had antioxidant activity.

## 4. Optimization of Culture Parameters for Enhancement of Microalgal Carotenoid Production

It has been reported that a wide variety of microalgae produce carotenoids. However, the quantity produced is quite small, which restricts commercial application. The isolation, screening, and selection of suitable algal candidates provide the foundation for the large-scale biosynthesis of carotenoids [92]. An additional component needed for better carotene synthesis is the selection of appropriate culture conditions for algal growth. The manufacturing of carotenoids would eventually be made easier by optimal growth. Additionally, a number of environmental stressors are thought to promote the storage of carotenoids [93].

### 4.1. Effect of Nutrient Conditions on Carotenogenesis

In order for microalgae to grow and flourish, carbon and nitrogen are crucial macronutrients. The carbon source may come from the atmosphere (CO2) or be synthesized by adding carbon-containing substrates such as glucose, glycerol, urea, etc. [94]. Microalgal cells’ potential to synthesize carotenoids can also be enhanced by growing medium with different ratios of carbon and nitrogen [92]. The addition of carbon substrate in media has been observed to increase astaxanthin concentration in *Chlorella zofingiensis*. The supplementation of carbon substrates, including citrate, malate, and pyruvate, under non-illuminated conditions accelerates the synthesis of astaxanthin [94].

In addition to playing a significant role in controlling the metabolism of microalgae, nitrogen is a key macronutrient in the synthesis of nucleic acids, proteinaceous compounds, and pigment molecules. The investigation of McClure et al. [95] has shown an enhanced accumulation of fucoxanthin contents in media supplemented with nitrogen. However, in another study, a nitrogen-starved conditions was also linked with increased production of canthaxanthin, as reported in *Chromochloris zofingiensis* and *Coelastrella* sp. [44,47,96]. The lack of nitrogen in the media acts as a stress factor for microalgal cells and may augment the synthesis of carotenoids [97]. The study of Pisal and Lele [98] has reported enhancement in β-carotene in the *Dunaliella salina* under nitrogen-limited conditions. The nitrogen-starved conditions increased β-carotene content from 1.65 pg/cell to 7.05 pg/cell.

However, various study authors reported that nitrogen limitation can lead to oxidative stress. In a study, Zhang et al. [99] reported nitrogen starvation lead to oxidative stress in *Chlorella sorokiniana*. Similarly, Çakmak et al. [100] reported limitation of nitrogen and supplementation of Zn significantly enhanced lipid peroxidation and H_2_O_2_ concentration in *Chlamydomonas reinhardtii*. Coulombier et al. [101] revealed the N-replete environments favor enhanced carotenoid production in *Nephroselmis* sp. and exhibited increased antioxidant activity. In contrast, N starvation condition decreased both total carotenoids and antioxidant activity.

As the proportions of carbon and nitrogen are closely linked with developmental characteristics, optimization of their concentration is an essential step to achieve the optimum concentration of carotenoids.

### 4.2. Effect of Light and Temperature on Induction of Carotenogenesis

Light and temperature are crucial factors for microalgal growth and biosynthesis of carotenoids. Different spectral ratios of light, such as red: far red, blue:red, green:red, and blue:green, have an impact on the relative pigment composition of microalgae [102]. The particular light pattern affects the productivity of microalgae. There is ample evidence supporting that light color and intensity cause microalgal cells to produce more carotenes [103].

The study of Hotos and Antoniadis [104] has suggested the contrasting role of light color and intensity on the bioproduction of carotenoids in *Phormidium* sp. and *Cyanothece* sp. The author reported an enhanced concentration of carotenoids in both the cyanobacterial strains under white low light conditions compared to high white light, red, green, and blue light after 18 days of growth. Similarly, Pisal and Lele [101] reported enhanced content of β-carotene per cell under white light and high light intensity. The higher irradiation from 800 lux to 2300 lux significantly enhanced carotenoid concentration. Indrayani et al. [105] reported a two-fold enhancement in carotenoid production in *Botryococcus braunii* high light intensity.

However, in contrast to the previous reports, Pelah et al. [34] reported enhanced production of astaxanthin in *Chlorella zofingiensis* at reduced light intensity. Similarly, McClure et al. [98] reported more than 4-fold enhancements in the fucoxanthin of *Phaeodactylum tricornutum* at low light intensity (100 μmol photons m^−2^ s^−1^) in comparison to high light intensity (210 μmol photons m^−2^ s^−1^). The authors reported that prolonged exposure to light might inactivate the enzymatic system participating in carotenoid synthesis and photo-inhibition of exposed algal cells [106,107]. In microalgal cells, the accumulation of carotenoids is significantly influenced by temperature.

In microalgal species, higher temperatures cause enhanced concentrations of carotenoids as a result of more photo-oxidative stress [107]. For example, the substantial effect of temperature on β-carotene synthesis in *Dunaliella salina* is reported by Zheng et al. [108]. Chekanov et al. [109] reported an increased concentration of astaxanthin in *H. pluvialis* at high temperatures. Similarly, García-González et al. [110] reported high temperature leads to enhanced accumulation of lutein *in Dunaliella salina*. However, several authors also reported high temperature-induced denaturation of enzymes involved in the synthesis of carotenoids [107]. Therefore, the authors recommended growing the microalgae at their optimum temperature range to achieve enhanced yields. Each of the strains has a different optimum temperature range [111].

As the carotenoids are present in both linear and cyclic form, the presence of methyl group around the isoprenoid causes steric hindrance, which favors *trans* isomer of carotenoids more than *cis* isomer. Usually, *trans* form has low solubility and high melting point than *cis* form [111,112]. Generally, most of the natural carotenoids are present in the thermo-stable trans form, and only a few are present in the *cis* form [113]. Authors reported that enhanced intensity of light and rise in temperature could cause the isomerization of carotenoids from *trans* to *cis* form [114,115]. These structural rearrangements have negative consequences on the physiological activity and nutritional constituency of carotenoids [116,117].

### 4.3. Effect of Salinity on Carotenoid Production by Microalgae

Salinity is one of the critical environmental factors governing biomass production and biochemical characteristics of microalgae. The optimum salinity level, however, varies among different species. Different investigators have shown varying concentrations of salt affecting the production of carotenoids. The most favorable salinity level for synthesizing the highest fucoxanthin content in the *Chaetoceros muelleri* has been observed at 45%. In contrast, the salt-tolerant *Amphora* sp. has demonstrated fucoxanthin production at an 85% salinity level [118]. Similarly, the strain *Isochrysis galbana* growing under 20% and 35% of salinity levels showed preferential growth and fucoxanthin production [119]. Nearly three folds augmentation of carotenoid production in *Desmodesmus* has been reported after exposure to 40% of NaCl with moderate illumination of light, as reported by Mehariya et al. [120]. The optimum NaCl concentration of 1% accelerates carotenoid accumulation in the strain *Scenedesmus* [121]. 

The role of halotolerant microalgal species *Dunaliella* in carotenoid production has been illustrated by number of researchers. Prieto et al. [122] have explored the potential of *Dunaliella salina* in carotenoid production under varying culture conditions involving batch, semi-continuous, open, and closed systems. The study demonstrated the best carotenoid productivity reaching up to 328.8 mg/L carotenoid culture in one month under a closed photobioreactor system. 

In microalgae, salinity stress can alter membrane permeability or damage the membrane, which affects the membrane’s integrity, fluidity, and ion transport selectivity. Therefore, high salinity stress results in decreased biomass synthesis as well as intracellular biomolecule synthesis [123,124,125]. Therefore, optimizing salt concentrations and selecting suitable microalgal species are essential steps toward the industrial production of desired carotenoids 

### 4.4. Heterotrophic and Mixotrophic Cultivation of Microalgae to Enhance Carotenogenesis

Heterotrophic and mixotrophic cultivation approaches have been commonly employed for microalgal biomass production concomitant with carotenoid synthesis [126,127]. The heterotrophic culture system relies on the dark supplementation of diverse carbon sources, including glucose, acetate, and glycerol [97,108]. On the other hand, mixotrophic cultivation refers to microalgal growth based on introducing different carbon-containing substrates under illuminated conditions. Under non-illuminated conditions (heterotrophic strategy), microalgal cells consume organic carbon sources supplemented in the medium resulting in favored growth and enhanced carotenoid synthesis. A heterotrophic cultivation strategy can potentially increase lutein biosynthesis [128]. Several investigations have suggested the incompatibility in the cultivation of some microalgae under heterotrophic conditions but augmented growth under a mixotrophic mode of cultivation. The growth and accumulation of carotenoids under heterotrophic and mixotrophic cultivation strategies also differ from species to species. The unfavorable growth of *D. salina* and β-carotene storage under heterotrophic cultivation and better accumulation of beta-carotene in mixotrophic culture conditions is well documented [129]. The alga *H. pluvialis* grew and accumulated astaxanthin under heterotrophic and mixotrophic culture conditions [130]. Shi et al. [128] have described the growth of *Chlorella pyrenoida* under heterotrophic conditions and reported enhanced lutein synthesis. Similarly, the production and accumulation of astaxanthin by *C. zofingiensis* under heterotrophic conditions supported by glucose as an external carbon source are noteworthy [37]. Thus, the two contrasting approaches for simultaneous growth and carotenoid production are substantially governed by the culture compatibility of algal species.

### 4.5. Cohesive Cultivation Approach for Enhanced Carotenoid Production by Microalgae

Cohesive cultivation, sometimes referred to as the co-culture or collaborative cultivation technique, is one of the crucial methods for the growth of microalgae and the production of carotenoids. This cultivation technique involves the simultaneous growth of more than two types of organisms with mutual benefits to each other in a reactor system. The simultaneous growth of the bacterium *Agrobacterium aurantiacum* and microalgae is reported in the previous study [130]. The microalgal cells derive oxygen from photosynthesis, which fulfills the requirement of oxygen for the growing bacterial cells. On the other hand, bacteria-produced carbon supports the growth of microalgae [131]. The growing bacterial cells may also exert stress, responsible for the possible enhancement of carotenoid synthesis in microalgae. A mutual advantage in cohesive cultivation can minimize the cost, and this strategy can be harnessed for the industrial production of carotenoids. However, the possible negative consequences of bacterially released toxic substances on microalgal cells induced cannot be overlooked [62]. If any, developing strategies for removing toxic metabolites and in situ detoxification could help eliminate the shortcomings. Another strategy for increasing carotenoid synthesis involves the accurate determination of concentrations that support microalgae in producing the required amount of carotenoids [132,133].

## 5. Advances in Carotenoid Production Using Biotechnological Approaches

The application of genetic and metabolic engineering can be used to enhance the production of desired metabolites, which has ceased due to the inherent limitation of metabolic capacity. As microalgae are a very diverse group and source of numerous metabolites, the latest biotechnological approach can be used as an opportunity to enhance the production [48]. Previously, with the help of mutagenesis and genetic engineering production of different microbial carotenoids has been enhanced [48,134]. Conventionally to improve the strains or to achieve a high yield of carotenoids, microalgal strains are subjected to mutagenesis [135]. Although different mutagens are used to develop the desired strains, mutagenesis via radiation is found to be a suitable approach to develop high-yield strains [136]. In a study, *Dunaliella bardawil,* after exposure to UV radiation induced the production of β-carotene in the mutant strain; similar results were reported with the strain of *Scendesmus* and *Chlorella* [137,138]. In another study, mutagen N-nitro-N-nitrosoguanidine has been reported to enhance astaxanthin production in *Haematoccus pluvialis* [139]. In a study, Yi et al. [140] use UV radiation to induce mutation in the *Phaeodactylum tricornutum* to enhance the production of carotenoids. Similarly, Sivaramakrishnan and Incharoensakdi [141] also used UV radiation to induce mutagenesis in *Scenedesmus* sp. to enhance the production of lipids. Further, Trovão et al. [142] briefly discussed random mutagenesis to improve the microalgal strain.

### 5.1. Metabolic and Genetic Engineering

Metabolic engineering refers to deliberate modulation in the metabolic pathway of an organism with the objective of producing desired molecules important as medicine, fuel, and pharmaceuticals for commercial application [143,144,145]. Metabolites are a diverse array of intermediate and final products in biosynthetic pathways. Few metabolites are known to regulate enzymatic activity [146]. Therefore, understanding the synthesis of cellular metabolites under given environmental conditions could help in manipulating the desired pathway in selected microalgae. The important information regarding the metabolites in terms of quality and quantity has been made quite possible by different forms of advanced mass spectroscopy in spite of considerable variations in chemical attributes, including molecular weight, polarity, volatile nature, and miscibility. So far, most of the microalgal metabolic engineering research have focused on lipid profiling under changing environmental parameters. Very limited information on carotenoid synthesis is available. The in situ investigation of single isolated cells using single-probe mass spectrometry could unravel the diversity of hidden metabolites more effectively under a given set of conditions [147]. This technique holds promise in future research rendered by minimized experimental errors and cellular complexities. Contrary to genomic and transcriptomic databases, the microalgal metabolite database is largely lacking, suggesting extensive research. Although metabolic models relying on the genome are available, however, experimental investigation-based metabolite information is limited. Some of the existing databases such as KEGG (https://www.genome.jp/kegg/pathway.html (accessed on 12 October 2022)), Reactome (https://reactome.org (accessed on 12 October 2022)), and Metacyc (https://metacyc.org (accessed on 12 October 2022)) containing useful metabolites information based on experiments as well as predictions may be considered for further exploration.

The process followed to enhance the microalgal carotenoid production by metabolic engineering is similar to the strategies followed in higher plants. This includes the modulation and regulation of enzyme biosynthetic pathways and formation of the metabolic sink, enhancing particular metabolite flux by interfering with their cellular metabolic process. However, this strategy needs extensive knowledge of biosynthetic enzymes and necessary information on how to modulate the cellular metabolism to enhance the flux [148].

The overexpression of particular enzymes, which can control the flux of particular products, or the enzyme present in the rate determination stage of the enzymatic reaction, can help achieve the enhanced desired metabolites without interfering with other metabolites. Thus, it is not mandatory to overexpress all the enzymes of the particular biosynthetic pathways to the same extent [149,150]. Although, it is not necessary that a single enzyme can control the flux of the particular desired metabolite. It is regulated through various enzymes by coordinated expression. Thus, during carotenoid synthesis, multiple enzymes must be overexpressed for enhanced production [151].

Manipulated expression of genes, including *PSY*, *PDS*, *BKY*, *OR*, *BCH*, and *LCY* under the control of different promoters, responsible for carotenoid synthesis, has been demonstrated in different algal species mostly belonging to *Chlamydomonas*, *Chlorella*, *Haematococcus*, and *Dunaliella* [152]. Studies have reported various microalgal strains such as *Chlorella zofingiensis* and *Haematococcus pluvialis,* and *Chlamydomonas reinhardtii*, whose gene has been modified for regulating carotenoid production [153]. Recent investigations have reported some genes and their metabolic pathways required to regulate carotenoid synthesis. For example, strains *Chlorella vulgaris*, *Chlamydomonas reinhardtii,* and *Volvox carteri* have regulatory enzymes such as phytoene synthase, encoded by the *PSY* gene responsible for the synthesis and regulation [154,155]. Similarly, strain *D. salina* contains two classes of *PSY* gene families’ upregulated during stress conditions, resulting in enhanced production of carotenoids [156]. Similarly, Couso et al. [157] reported upregulation of carotenoid synthesis in the *Chlamydomonas reinhardtii* under stress. Vidhyavathi et al. [158] reported altered expression of the carotenoids synthesis gene in the *Haematococcus pluvialis* strain under nutrient-stress conditions.

The principle behind any cell’s metabolic engineering is to understand the biochemical pathway of the cell in order to manipulate biochemical steps through modifying enzymatic activity, which leads to the creation of a sink, enhancing the flux to produce required metabolites [159,160,161]. The overexpression of the gene directing the synthesis of a particular enzyme leads to the production of the desired metabolite, not interfering with other metabolite production. Therefore, only the enzyme of the final step of any biochemical pathway required for the formation of the desired product may be taken into account for overexpression. For example, overexpression of the gene leading to the synthesis of the PDS enzyme, a rate-limiting step, is crucial for ζ-carotene production [162]. The role of different genes involved in carotenoid production is presented in Table 3.

Nuclear overexpression of endogenously mutated *PDS* gene in the chloroplast may lead to enhanced astaxanthin production. A study conducted on *Chlorella zofingiensis* and *Haematococcus pluvialis* by performing overexpression of nuclear mutated endogenous gene PDS resulted in enhanced astaxanthin content by 26% and 32%, respectively [148,149]. A similar study was conducted in the chloroplast of *Haematococcus pluvialis*, which showed a 90% enhancement in astaxanthin content [150]. Moreover, it is a coordinated action of multiple enzymes whose overexpression results in the production of specific carotenoids. Even the downregulation of specific enzymes has helped to overexpress specific enzymes [151]. For example, in a study, the downregulation of the LCYE gene led to the overproduction of β-carotene. A study also reported that the ZEP gene knockout mutant, achieved through CRISPER-CAS9 in *Chlamydomonas reinhardtii,* resulted in higher zeaxanthin content than the wild type [178].

Further study showed the integration of the bkt gene of *Haematococcus pluvialis* by using metabolic engineering in the chloroplast of *Dunaliella salina*, which leads to the production of astaxanthin [179]. However, the overproduction of several carotenoids is obstructed through feedback inhibition and encountered overexpression of feedback-resistant enzymes. The metabolic sink is considered a reservoir of specific metabolites, which are transported to the sites, and the overproduction of specific metabolites is inhibited through feedback inhibition. However, it has been observed that in microalgae, *Haematococcus* lipid act as a metabolic sink that regulates carotenoid production [180,181]. Similarly, Rabbani et al. [182] reported that the triacylglycerol deposition induced the β-carotene synthesis in *Dunaliella bardawil*.

### 5.2. Selection of Hosts and Transformation Methods

The strategies of transformation play a significant role in the genetic manipulation of microalgae to achieve the desired products. Successful transformation based on electroporation, biolistic approach, polyethylene glycol, silicon carbide whiskers, glass bead, and conjugation been reported in the numbers of microalgae [183,184]. In addition, the selection of transformed cells involves the application of selectable markers, including resistance to antibiotics, herbicides, and auxotrophic markers. The selection of specific transformation techniques and markers, however, may vary from species to species. The considerable diversity in the cellular system of microalgae, nevertheless, limits the usefulness of microbe and plant-derived markers for selection [185]. However, such as other mechanisms, transformation techniques also have some merits and demerits. Such as rapid growth, reduced risk of contamination, uniformity (single cell microalgae proteins) in the products of microalgae has advantages over the plant system [186]. But the lesser efficacy of carotenoids productions in microalgal cells has been considered a a major disadvantage in comparison to plant systems. During the transformation, selection and identification of the host cell that will have incorporated with foreign genes and their ability of expression have been considered as a prime factor for successful transformation. The host structure and method of transformation also have a prominent role in transformation [187]. A general screening of transformants has been carried out with the addition of antibiotics or herbicides in the media, so optimization of culture media and sensitivity against the antibiotics or their concentration must be predefined [188,189]. The first successful nuclear transformation has been reported in the *Chlamydomonas reinhardtii*, a single-cell green alga and also considered a model organism because of its well-studied genomes [190]. Initially, the transformation has been executed using the biolistic approach, glass bead agitation, and electroporation [191,192,193,194]. In the recent past, various authors used transformation techniques to overexpress particular genes in microalgae. For example, Steinbrenner and Sandmann [150] improve the biosynthesis of astaxanthin by the transformation of phytoene desaturase in *Haematococcus pluvialis*. Kumari et al. [195] overexpressed the plant’s *Brassica oleracea* “OR” gene in *C. reinhardtii*. Simon et al. [163] reported enhanced production of violaxanthin and zeaxanthin after transforming the BCH gene of *Chlamydomonas reinhardtii* in *Dunaliella salina.*

In spite of substantial achievement in transformation, low efficiency has been recorded in most algae except *Chlamydomonas* in comparison to plants. The direct transfer of bacterial vectors into diatom through conjugation has emerged as an efficient approach to transformation [184]. The technique bears multiple benefits over traditional methods, including the transfer of large-sized genomes, natural replication of episomal vectors, deletion of the transferred gene in the absence of selection pressure, and minimal chances of epigenetic influences [196]. Studies have presented the generation of trans-gene-free mutants in *P. tricornutum* and *Nannochloropsis oceanica* using conjugation-based genome editing mediated by CRISPR/cas9 [197,198]. The transformation efficiency was twenty to a hundred times greater. However, mutant visibility in transformants was considerably delayed owing to reduced Cas9 expression [197]. Therefore, successful transformation in suitable microalgal species relying on available methods is an important strategy for the enhanced production of desired carotenoids. Further improvements in this direction would raise the productivity of carotenoids for commercial application.

## 6. Commercialization and Policy Concerns

As microalgae are a rich source of food, cosmetics, and high-value products, the commercialization and availability of their products at a reliable price are needed for success. There are numerous synthetic carotenoids already present in the market, therefore, it has become important to extract natural carotenoids, which must be economically cheap, easy to availability, and of fewer side effects. However, these microalgal products are consumed by humans for health and nutrition, these products are subjected to a range of rule regulations of international standards, and before entering the market, these products must full fill these standard regulations. Although these regulations vary from country to country [199], these issues have been well described in the previously published paper [200,201,202].

The genetic manipulations have been considered as one of the vital tools to enhance the production of microalgal products. Therefore, it is mandatory to evaluate the safety concern of genetically modified (GM) algae after consumption and their impact on consumers and environmental health. It has been found that the cultivation of genetically modified microalgae may cause the risk of uncontrollable spills. These GM algae, after growth, may compete with the natural species and exclude them. In addition, also becomes a threat of horizontal gene transfer, interbreeding, and genetic contamination. However, the algal blooms and uncontrolled or unpredictable properties of GM traits are some severe challenges for GM algae [203]. In order to avoid the invasion of foreign strains into the non-native local environment, it should be controlled and monitored by the regulatory bodies [204]. Further, there is a need to follow a strict monitoring policy to handle the cultivation process, and also an in-depth study is required to control the negative consequences on the health and environment [185,205].

## 7. Future Perspective and Conclusions

Microalgae are an important source of valuable pigment molecules, including carotenoids, harboring antioxidant and coloring characteristics. Diverse microalgae are listed to produce varying concentrations of carotenoids such as vialoxanthin, lutein, astaxanthin, α and β-carotene, zeaxanthin, fucoxanthin, and neoxanthin depending upon the environmental conditions. The antioxidant properties of carotenoids responsible for free radical quenching can be harnessed for therapy of human diseases, therefore, are suggested as part of a healthy diet. For instance, carotenoids are known to possess anticancer and antitumor activities apart from characteristic contributions in the regulation of eye disorders and improvement of cardiovascular disorders. Under natural conditions, the amounts of carotenoid produced by microalgal cells are too low to fulfill the requirement. Further, the content of carotenoid synthesized varies considerably from species to species. The amount of biologically synthesized carotenoids can be enhanced by manipulating culture conditions such as nitrogen, carbon, light intensity, color, temperature, and salinity. The selection of suitable microalgal candidates, along with strain improvement using CRISPR/Cas9 technology [32], is another important step toward the production of intended carotenoids. Modern approaches falling in the category of metabolic and genetic engineering can be employed for the industrial production of biologically important carotenoids.

So far, only limited numbers of valuable carotenoids are commercialized, and many more are still to be discovered from a very large pool. Further explorations of carotenoids with rare occurrences, together with the currently isolated ones, are important hotspot areas of research. For example, the carotenoid “myxol” with antioxidant activity higher than β-carotene has been isolated from cyanobacteria *Oscillatoria limosa*, *Anabaena variabilis,* and *Nostoc commune* [206]. Identification of newer carotenoids and their utilization as food and human health applications is an emerging research area. All of the known carotenoids have common precursors. The natural synthesis of carotenoid precursors within microalgae could be an important breakthrough in the industrial production of chemically synthesized carotenoids, thus minimizing the cost of overall production [50]. Microalgal biomass processing for maximum extraction of carotenoids is regarded as one of the major challenging stages. The application of suitable technology for biomass harvesting, cell disruption and solvents for extraction as well as purification in a natural state at the minimum expense is, therefore, a preliminary step for the commercialization of carotenoids. In this context, the search for non-toxic switchable solvents based carotenoid extraction methods without affecting the bioactivity is attractive for large-scale production.

However, for sustainable production, there should be widespread bio-prospection of microalga species is needed which have higher growth rates and better capability of carotenoid production [207]. In addition, the wastewater originating from different industries can also be used as a source of nutrients during microalgal biomass production, indicating opportunities in low-cost production. But prior to mass cultivation, the level of hazardous heavy metals, pesticides, and emerging contaminants should be investigated thoroughly. The identification and expression of suitable candidate genes using suitable vectors directing the synthesis of particular carotenoids in the compatible microalgal host is an important step for industrial production. A detailed understanding of biochemical routes culminating in the synthesis of the desired carotenoid could help in enhancing microalgal carotenoid production.

## Figures and Tables

**Table 1:** The chemical structure of some common microalgal carotenoids.

Carotenoid	Chemical Structure
Astaxanthin	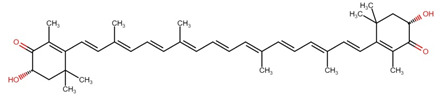
β-carotene	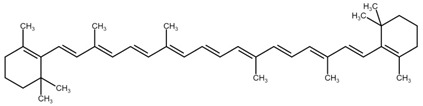
Lutein	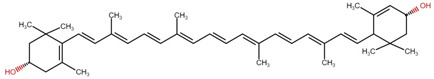
Zeaxanthin	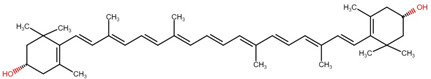
Canthaxanthin	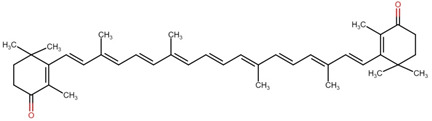
Violaxanthin	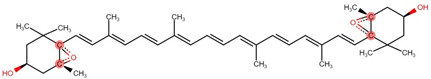
Lycopene	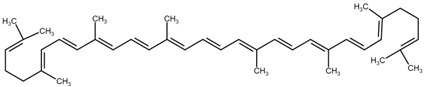

**Table 2:** Production of different types of carotenoids from microalgae and their properties.

Microalgae	Carotenoids	Isolated From	Yield	Properties	Reference
*Chlorella zofingiensisis*	Astaxanthin; canthaxanthin	-Not mentioned	11.70 mg/g	Nutraceuticals foods with Anticarcinogenic and antioxidant properties	[34]
*Dunaliella salina* CS-265; *Tetraselmis suecica* CS-187	Lutein, zeaxanthin, astaxanthin, neoxanthin, violaxanthin, and β-carotene	Alice Springs, NT, Australia, and Brest, France	8.87 mg/g	Carotenoids increase microalgae; affect growth and development in plants through environmental stress.	[35]
*Coelastrella* *striolata* var. multistriata	canthaxanthin, astaxanthin and β-carotene	Shizuoka, Japan	56.0 mg/g	Antioxidant potential in lipid foods; feed supplements, natural antioxidants and food dyes.	[36]
*Chlorella zofingiensis*	Astaxanthin	Rockville, USA	10.3 mg/g	Enhanced biosynthesis of secondary carotenoids	[37]
*Scenedesmus* sp. strain CCALA 1074	Lutein	Plankton of the Sihl river; Switzerland	19.70 mg/L/day	Antioxidative and anti-inflammatory properties	[38]
*Chlorella zofingiensis* mutant (CZ-bkt1)	Zeaxanthin, lutein, and β-carotene	American Type Culture Collection 60 (ATCC, Rockville, MD, USA)	7.00 mg/g 13.81 mg/g 7.18 mg/g	Microalgae accumulate high amounts of zeaxanthin.	[39]
*Parachlorella* sp. JD-076	Lutein	Freshwater, South Korea	4.95 mg/g	Enhanced dry cell weight and lutein productivity; alleviating cardiovascular diseases	[40]
*Tisochrysis lutea*	Fucoxanthin	Tropical region of the Pacific	9.81 mg/L/d	Antioxidant, anti-obesity, and anti-diabetic	[41]
*Odontella aurita*	Fucoxanthin	University of Copenhagen	7.96 mg/L/d	High levels of eicosapentaenoic acid; pigments, fibers and phytosterols	[42]
*Phaeodactylum tricornutum,*	Fucoxanthin	Not mentioned	26.1 mg/g	Anti-inflammatory, antioxidative and antiproliferative effects	[43]
*Chlorella zofingiensis*	Astaxanthin	University of Texas Culture Collection of Algae (UTEX, Austin, USA)	4.89 mg/g	Induced the accumulation of neutral lipids, especially TAG and astaxanthin	[44]
*Phormidium autumnale*	β-carotene, zeaxanthin, lutein, echinenone	Cuatro Cienegas desert, Mexico	107,902.5 kg/year	Antiviral, Antibacterial, Anticancer, and Antifungal Properties	[45]

**Table 3:** Bioengineering approaches enhance the production of carotenoids by overexpression of the genes.

Microalgae	Carotenoids	Gene	Response	Reference
*Dunaliella salina*	Violaxanthin; Zeaxanthin	*bch*	Accumulation of β-carotene; conversion of β-carotene to xanthophylls	[163]
*Haematococcus pluvialis* 34-1n	β-carotene and β-carotene	*bkt* and *bch*	Catalyzing the biosynthesis of astaxanthin in transgenic algae	[164]
*Phaeodactylum tricornutum*	Fucoxanthin and β-carotene	*by*	Improve expression; enhanced green fluorescent protein gene	[165]
*Haematococcus pluvialis*	β-carotene zeaxanthin and adonixanthin	*bkt*	Accumulation of astaxanthin was shown in *E. coli*	[166]
*Chlorella zofingensis*	Astaxanthin	*psd*	Molasses triggered the up-regulation of genes involved in fatty acid and also astaxanthin biosynthesis	[167]
*Chlamydomonas* *reinhardtii*	β-carotene, α-carotene, lutein and violaxanthin	*or*	Increase carotenoid accumulation in OR-overexpressing algae	[168]
*Phaeodactylum tricornutum*	-	*glut1*	Conversion of an obligate photoautotrophic organism	[169]
*Chlorella zofingiensis*	β-carotene	*ctrO*	High enzymatic activity of converting zeaxanthin to astaxanthin via adonixanthin	[170]
*Chlamydomonas* *reinhardtii*	ζ-carotene	*PDS*	Enhanced desaturation activity; promoted norflurazon resistance	[171]
*Chlorella zofingiensis*	Violaxanthin and Lutein	*by*	Enhancement of carotenoids biosynthesis	[172]
*Chlorella (Chromochloris) zofingiensis*	α-carotene and lutein	*lcy-e*	Light and nitrogen starvation stresses enhanced the accumulation of carotenoids significantly	[173]
*Chlamydomonas reinhardtii* strain CC-4102	Canthaxanthin	*CrBKT*	Production of ketolation product from β-carotene; reduction in the chlorophyll concentration	[174]
*Chlamydomonas* *reinhardtii*	β-carotene	*crtYB*	Increase in the lutein yields and β-carotene	[175]
*Dunaliella Parva*	β-carotene	*by*	Enhanced the antioxidant activity of carotenoids	[176]
*Nannochloropsis oceanica*	β-carotene	*PDS*	Effect on carotenoid profiles; carotenoid biosynthesis in *N. oceanica*	[177]

## Data Availability

Not applicable.
